# Oocyte Degeneration After ICSI Is Not an Indicator of Live Birth in Young Women

**DOI:** 10.3389/fendo.2021.705733

**Published:** 2021-08-16

**Authors:** Xiaokun Hu, Yuliang Liu, Xiubing Zhang, Pingyin Lee, Yangxing Wen, Chenhui Ding, Canquan Zhou, Yanwen Xu

**Affiliations:** ^1^Reproductive Medicine Center, The First Affiliated Hospital of Sun Yat-sen University, Guangzhou, China; ^2^Guangdong Provincial Key Laboratory of Reproductive Medicine, The First Affiliated Hospital of Sun Yat-sen University, Guangzhou, China

**Keywords:** oocyte degeneration, intracytoplasmic sperm injection, cumulative live birth rate, ovarian stimulation, infertility

## Abstract

**Introduction:**

Intracytoplasmic sperm injection (ICSI) was introduced in 1990s as one of the most dramatic breakthroughs in assisted reproductive technology. Even with advances in ICSI technology, this mechanical micromanipulation carries a 5 to 19% risk of oocyte degeneration. Whether the presence of oocyte degeneration reflects the sibling oocyte quality and predicts the sibling embryo development potential and clinical pregnancy outcomes remains controversial. There is no study showing the competence of the sibling embryos from the prospective of cumulative live birth rate. Whether oocyte degeneration is associated with poor quality of the remainder of the cohort remains further to be elucidated.

**Method:**

This retrospective observational study included a total of 488 OPU cycles underwent ICSI with fresh cleavage stage embryo transfer and successive frozen/thawed embryo transfer (FET) cycles from January 2018 to December 2019. All female patients were under the age of 35 years, and underwent ICSI with or without oocyte degeneration (OD). Cycles with at least one oocyte degenerated were defined as oocyte degeneration group (OD group), and cycles with no oocyte degenerated were defined as non-OD group. The OD group was further divided to three subgroups according to different oocyte degeneration rate (<10%, 10-20%, and >20%).

**Results:**

There were no significant differences with regards to implantation rate (38.5% *vs* 35.1%, *P*=0.302), clinical pregnancy rate (54.9% *vs* 50.3%, *P*=0.340), and LBR per OPU cycle (47.0% *vs* 42.9%, *P*=0.395) between OD and non-OD groups. Initial gonadotropin dosage, E_2_ level on hCG day and the number of matured oocytes appeared to be independent risk factors for OD. The adjusted odds ratio of live birth rate per OPU cycle were similar in different oocyte degeneration rate subgroups. The ongoing pregnancy/LBR per transfer in FET cycles was not significantly different between OD group and non-OD groups (38.8% *vs* 43.9%, *P*=0.439). The cumulative LBR per OPU cycle was also comparable between OD and non-OD group (63.4% *vs* 64.8%, *P*=0.760).

**Conclusion:**

The results provide cycle-based evidence that the presence of oocyte degeneration after ICSI is not an indicator for predicting the cumulative live birth rate per OPU cycle in young women.

## Introduction

Intracytoplasmic sperm injection (ICSI) was introduced in 1990s as one of the most dramatic breakthroughs in assisted reproductive technology (ART). Even with advances in ICSI technology (1–4), this mechanical micromanipulation carries a 5 to 19% risk of oocyte degeneration (5, 6), resulting in reduction of the numbers of survived oocytes and embryos. The degeneration can be observed either immediately after withdrawal of the injection needle, coexisting with sudden breakage on needle entry and leakage of cytoplasm (7), or following normal injection, identified by a retracted and/or darken ooplasm on the following day.

Limited investigation has addressed the causes of oocyte degeneration. Mechanical factors, such as sudden and/or difficult breakage of the oolemma (5, 8), and absence of persistence of funnel after withdrawal of the injection pipette (9) have been reported as events possibly favoring oocyte degeneration during ICSI. Oocyte factors, such as oocyte cytoplasmic viscosity (9, 10) and oolemma fragility (11), had been investigated too. A multivariate analysis showed that oocyte degeneration is neither technician nor physician dependent, but is likely a function of the inherent oocyte quality (12). However, whether the presence of oocyte degeneration reflects the sibling oocyte quality and predicts the sibling embryo development potential and clinical pregnancy outcomes remains controversial. A retrospective study (13) deduced that oocyte degeneration may be associated with decreased embryo quality for embryo development kinetics was disturbed. However, no differences in clinical outcomes such as implantation rate or clinical pregnancy rate (CPR) were found in fresh embryo transfer cycles in retrospective studies (11, 13). Whether the presence of oocyte degeneration is associated with poor quality of the remainder of the cohort remains a question. To the best of knowledge, there is no study showing the competence of the sibling embryos from the prospective of cumulative live birth rate. Therefore, it is definitely worthy of further investigation.

In this study, we included ICSI cycles with fresh cleavage stage embryo transfer in the young women, stratified by oocyte degeneration rate (ODR), for the purpose of investigating the cumulative LBR from both the fresh ET and the successive FET cycles, thus to fully evaluate developmental potential and capacity of the same cohort of embryos. In addition, confounding variables that may account for oocyte degenerated (OD) after ICSI were investigated.

## Materials and Methods

### Study Participants

This was a retrospective cohort study, including all the oocyte retrieval cycles from young women who underwent ICSI from January 2018 to December 2019 at the Reproductive Medicine Center of the First Affiliated Hospital of Sun Yat-sen University. The inclusion criteria were as follows: female age was younger than 35 years; the first or second oocyte retrieval cycles were performed between January 2018 and December 2019; the number of oocyte retrieval was between 8 and 20; all the cycles performed fresh embryo transfer on day 3 after insemination. Cycles with at least one oocyte degenerated after ICSI were defined as the oocyte degeneration group (OD group), and cycles with no oocyte degenerated after ICSI were defined as the non-OD group. The OD group was further divided to three subgroups according to different oocyte degeneration rate (<10%, 10-20%, and >20%) compared with non-OD group (oocyte degeneration rate 0).

### Stimulation Protocol, ICSI, Embryo Culture, and FET

All the patients underwent controlled ovarian stimulation according to our routine protocols, including mid-luteal phase long protocol and antagonist protocol, as previously described in details (14). Recombinant FSH (Gonal-F, Merck-Serono) or a combination of rFSH with hMGs (Menopur, Ferring Pharmaceuticals) was used for controlled ovarian stimulation. Dosages were individualized for each patient according to the patient’s age, weight, and ovarian reserve, and further adjusted according to serum estradiol levels and vaginal ultrasound measurement of follicular diameter, obtained every two or three days. Ovulation was triggered using 250 μg of recombinant hCG (Ovidrel; Merck-Serono) or 5,000–10,000 IU hCG when two follicles reached 18 mm or three follicles reached 17 mm in diameter.

**Figure 1:**
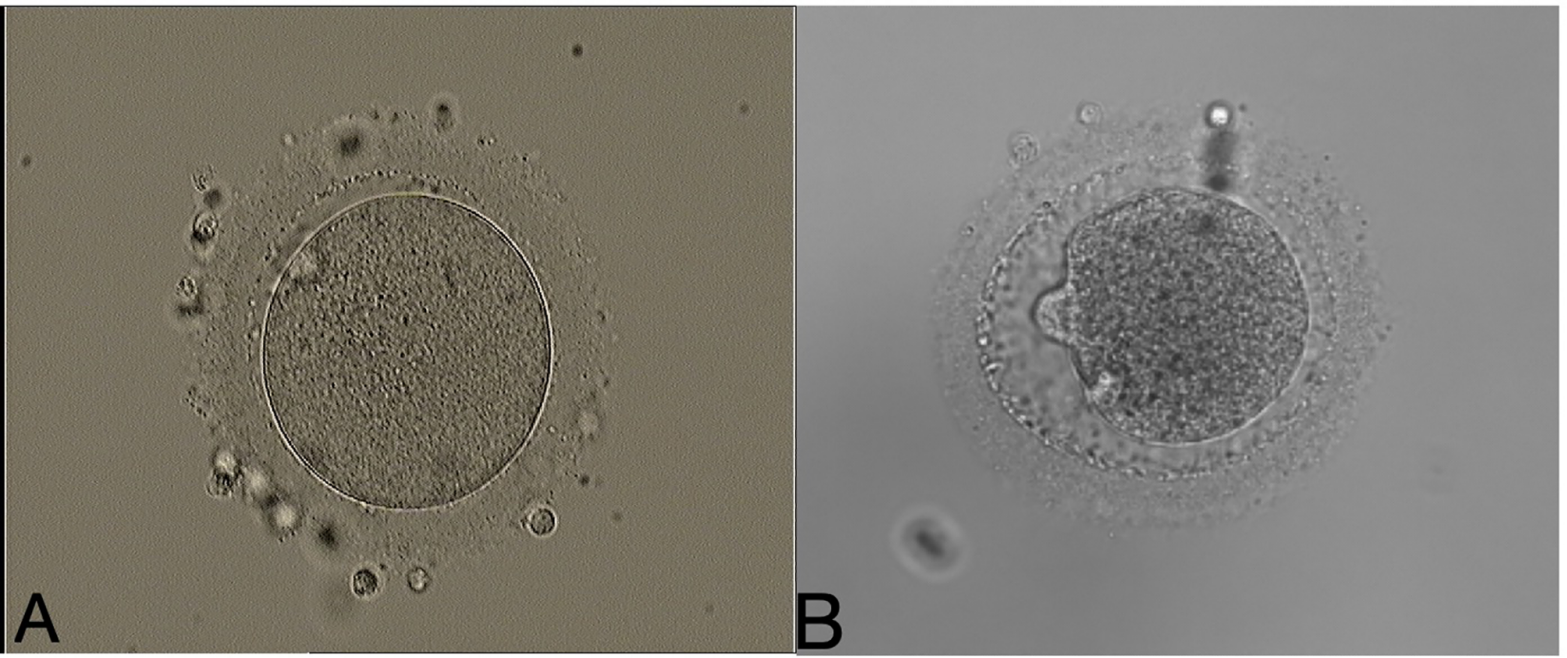
Example images of a matured oocyte (MII oocyte), and degenerated oocyte after ICSI. **(A)** A matured oocyte (MII oocyte). **(B)** Degenerated oocyte after ICSI. (200×magnification).

Transvaginal ultrasound-guided oocyte retrieval was performed 34–36 hours later. Cumulus–oocyte complexes were isolated and cultured in IVF fertilization medium (Vitrolife, Gothenburg, Sweden) at 37°C in 6% CO_2_ for 2–4 h. After ovum pick-up, the cumulus and corona cells of oocyte-cumulus complexes were removed using 80 U/ml hyaluronidase solution (VitroLife, Gothenburg, Sweden) and mechanical disruption with denudation pipettes (RI Consumables). The time of oocytes exposed to the hyaluronidase enzyme is less than 20 seconds in our lab. Mature oocytes (MII oocytes) were identified and picked up for subsequent ICSI insemination. It is generally recognized that a “normal” human MII oocyte should have a round, clear zona pellucida (ZP), a small perivitelline space (PVS) containing a single not fragmented first polar body, and a pale, moderately granular cytoplasm with no inclusions. The collected mature oocytes were rinsed with G-MOPS medium containing 5% HSA and incubated in G-IVF medium with 10% HSA at 37°C in 6% CO_2_, 5% O_2_, and 89% N_2_ for 2 to 3 hours before ICSI. ICSI was performed in our lab under strictly controlled air purification system. More than 1,000 ICSI cycles were performed each year. Single sperm was selected for injection into the oocytes through injection pipettes (OD:5.5um, The Pipette Company, Australian). Oocytes were denuded between 2 to 4 hours post collection. ICSI will be performed right after denuding in our lab, and the mean time interval between denuding and injection was less than 1 hour. The meiotic spindle was not visualized during ICSI procedure in clinics. When doing ICSI, the polar body is placed in the 12 o’clock or 6 o’clock position, ﻿and the injection pipette is inserted from the 3 o’clock position. Theoretically, the location of cytoplasm aspirated from a zona situated far from the meiotic spindle. The average time taken for injecting an oocyte was less than 2 minutes. After injection, the inseminated oocytes were incubated in G1 medium containing 5% HSA at 37°C in 6% CO_2_, 5% O_2_, and 89% N_2_. Fertilization and oocyte degeneration were assessed under the invert microscope 16–18 h later after ICSI. Oocyte degeneration ﻿is often characterized by oocyte lysis after withdrawal of the injection needle or being noted the next day by retracted and/or darkened ooplasm. Images of matured oocytes (MII oocytes), degenerated oocytes after ICSI were shown in **Figure 1**. After 2 days culture in G1 medium, cleavage stage embryos would be moved to G2 medium containing 5% HSA at 37°C in 6% CO_2_, 5% O_2_ and 89% N_2_ for an additional 2–3 days of blastocyst culture.

No more than two cleavage stage embryos were transferred on the morning of the 3th day after oocyte retrieval. According to our embryo culture policy, if there were no more than two cleavage stage embryos available after fresh embryo transfer, they will be vitrified on day 3, otherwise, all the rest will be performed blastocyst culture. Surplus cleavage stage embryos or blastocysts were vitrified using the Cryotop (Kitazato Supply Co., Fujinomiya, Japan) method (15) for subsequent FET cycles if necessary. Embryo morphology was evaluated according to the Istanbul Consensus Workshop on Embryo Assessment (16).

For FET cycles, endometrial preparation protocols included hormone therapy (HT) cycles, for patients with irregular menses, and natural cycles (NC), for patients with regular menses, as previously described in details (17). Cleavage stage embryos were transferred on the 4th day after progesterone administration or on the 3th day after ovulation. Single blastocyst was transferred on the 6th day after progesterone administration or on the 5th day after ovulation. All embryo transfers were performed under transabdominal ultrasound guidance. Serum hCG levels were determined 12–14 days after embryo transfer. Progesterone and estrogen would be continued for another 3 weeks until a clinical pregnancy was confirmed by transvaginal ultrasound. Luteal support would be stopped on the 10th week of gestation. Patients were followed from their first fresh cycles either to the first live birth or until November 2020, regardless of whether all frozen embryos had been transferred.

### Outcome Measures and Definitions

Oocyte degeneration rate is calculated by the number of degenerated oocytes after ICSI on Day 1/number of MII oocytes collected on Day 0. Normal fertilization rate is calculated by the number of normal fertilized oocytes on Day1(2PN)/number of MII oocytes collected on Day 0, including degenerated oocytes. In our clinic, one to two top quality embryos were transferred on day 3 after oocyte retrieval, and the rest would be cultured to blastocyst stage. Therefore, blastocyst formation rate is calculated as number of blastocyst on Day5/6 divided by number of cultured surplus cleavage stage embryos on Day3.

Serum β-hCG levels were tested on the 14th day after cleavage stage embryo transfer. Follicular output rate (FORT) was calculated as the ratio between the number of pre-ovulatory follicles and antral follicle count (AFC). A clinical pregnancy was defined as the presence of one or more gestational sacs. Biochemical pregnancy was defined as pregnancy that was diagnosed only by the detection of hCG in serum or urine but failed to develop into a clinical pregnancy. The implantation rate was defined as the number of gestational sacs visualized on transvaginal ultrasound divided by the total number of embryos transferred. The miscarriage rate was calculated as the number of pregnancy failures after a gestational sac had been documented by transvaginal ultrasound divided by the total number of clinical pregnancies. Any pregnancy that went beyond 20 weeks of gestation was considered an ongoing pregnancy. Monozygotic twins that resulted from a single embryo transfer were counted as one implantation or one ongoing pregnancy or live birth.

All pregnancies were followed up by our staffs until the end of the gestation.

### Statistical Analysis

Data analysis was performed with the IBM Statistical Package for Social Sciences (SPSS) version 22.0. Categorical data were presented as number and percentage. Continuous variables were given as mean±SD. Categorical data were analyzed using Fisher’s exact test or *χ*2 test and continuous variables were analyzed using a Student *t* test. Odds ratios (ORs) with 95% confidence intervals (CIs) was reported. One factor analysis of variance (one-way ANOVA) was applied for the comparison between groups for continuous data. Homogeneity of variances was tested and Student–Newman–Keuls (SNK) test was used with equal variances. When the variances were unequal, Tamhanes T2 test was applied as indicated. Potential confounder adjustment was performed using multivariable regression analysis. A *P*-value was considered statistically significant if <0.05, followed by pairwise comparisons (corrected for multiple comparisons using the Bonferroni method) whenever significant.

## Results

### Baseline Characteristics of Study Population and Risk Factors of OD

Overall demographics including baseline ICSI characteristics, ovarian stimulation parameters and perinatal outcomes of the study cohort are presented in **Supplemental Table 1**. A total of 488 OPU cycles with fresh cleavage stage embryo transfer were included. The overall oocyte degeneration rate was 4.64%, and the live birth rate per OPU cycle was 44.26%.

**Table 1** presents the comparison between OD group and non-OD group. No differences were observed in the two groups with regards to female/male age, BMI, basal FSH level, basal LH level, basal E_2_ level, total gonadotropin dosage. The OD group had better ovarian response with a significantly higher E_2_ level on hCG day (*P*=0.014) and a higher number of matured oocytes (*P*=0.005). In addition, the follicle output rate obtained from OD group was 59.0%, significantly higher than 51.2% obtained from non-OD group (*P*=0.000). Although oocyte maturation rate in OD group was significantly higher than that in non-OD group (84.2% *vs* 80.3%, *P*=0.000), the normal fertilization rate in the OD group was significantly lower than in the control group (69.4% *vs* 80.8%, *P*=0.000). Furthermore, the OD group had a statistically significantly lower numbers of normal cleavage embryos, available embryos, and good quality embryos (*P*=0.044, *P*=0.032, *P*=0.009, respectively) than the non-OD group. However, there were no significant differences in the blastocyst formation rate, implantation rate, clinical pregnancy rate, and live birth rate per OPU cycle between the two groups.

**Table 1:** Baseline characteristics and reproductive outcomes of fresh cycles between OD group and Non-OD group.

Characteristic	None-OD group	OD group	*P*
	N = 324	N = 164	
Female age (y)	30.50 ± 3.67	30.85 ± 3.81	0.325
Male age (y)	33.31 ± 5.86	33.65 ± 6.18	0.555
History of infertility	3.73 ± 2.29	3.66 ± 2.76	0.775
Basal FSH, IU/L	5.62 ± 1.39	5.80 ± 1.32	0.173
Basal LH, IU/L	3.43 ± 2.07	3.18 ± 1.44	0.175
Basal E2, pg/ml	31.15 ± 22.19	31.12 ± 12.65	0.989
Female BMI (kg/m2)	21.27 ± 2.68	21.37 ± 3.13	0.699
Stimulation protocol			
mid-luteal phase long protocol	68.2 (221/324)	73.2 (120/164)	0.259
antagonist protocol	31.8 (103/324)	26.8 (44/164)	–
Total gonadotropin dosage	2241.71 ± 861.49	2375.15 ± 787.33	0.097
LH level on hCG day, IU/L	1.21 ± 1.18	1.17 ± 0.95	0.740
E2 level on hCG day, pg/ml	2456.80 ± 939.10	2682.15 ± 955.73	0.014
P level on hCG day,	0.71 ± 0.27	0.75 ± 0.26	0.220
Endometrial thickness on hCG day (mm)	11.32 ± 2.14	11.44 ± 3.61	0.701
No. of retrieved oocytes	13.11 ± 3.35	13.57 ± 3.38	0.157
No. of matured oocytes	10.53 ± 3.31	11.42 ± 3.26	0.005
No. of normal fertilized oocytes	8.51 ± 3.21	7.92 ± 3.08	0.052
No. of normal cleavage embryos	8.27 ± 3.17	7.66 ± 3.02	0.044
No. of D3 embryos for blastocyst culture	5.53 ± 3.43	4.73 ± 3.13	0.013
No. of vitrified blastocysts	3.31 ± 2.76	2.80 ± 2.62	0.052
No. of available embryos	4.87 ± 2.31	4.41 ± 2.13	0.032
No. of good quality embryos	4.05 ± 2.51	3.44 ± 2.23	0.009
No. of transferred D3 embryos	1.91 ± 0.29	1.93 ± 0.25	0.395
No. of implanted D3 embryos	0.67 ± 0.75	0.74 ± 0.76	0.304
FORT	51.2 (2009/3925)	59.0 (1103/1870)	0.000
Oocyte maturation rate	80.3 (3412/4248)	84.2 (1873/2225)	0.000
Normal fertilization rate	80.8 (2758/3412)	69.4 (1299/1873)	0.000
Normal cleavage rate	97.1 (2679/2758)	96.8 (1257/1299)	0.519
Blastocyte formation rate	59.9 (1073/1791)	59.3 (460/776)	0.764
Implantation rate	35.1 (217/619)	38.5 (122/317)	0.302
Biochemical pregnancy rate	4.9 (16/324)	3.0 (5/164)	0.331
Miscarriage rate	12.9 (21/163)	10.0 (9/90)	0.497
Ectopic pregnancy rate	1.2 (2/163)	3.3 (3/90)	0.249
Clinical pregnancy rate	50.3 (163/324)	54.9 (90/164)	0.340
Live birth rate/per OPU cycle	42.9 (139/324)	47.0 (77/164)	0.395
Multiple pregnancy rate	23.9 (39/163)	27.8 (25/90)	0.500

Values are mean ± SD or percentage (number); BMI, body mass index; E2, estradiol; FSH, follicle stimulating Hormone; LH, luteinizing hormone; FORT, follicle output rate.

**Figure 2:**
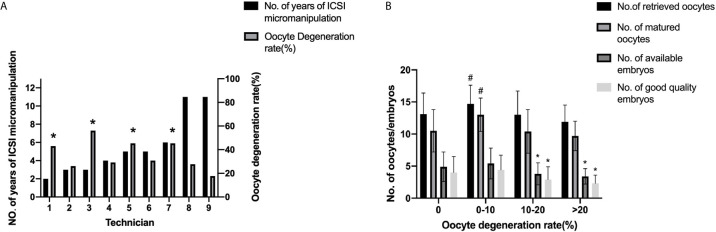
Oocyte degeneration rates among ICSI technicians and fresh cycle outcomes according to different oocyte degeneration rates. **(A)** Oocyte degeneration rates among ICSI technicians. *P<0.05 compared with the 9^th^ technician. **(B)** Fresh cycle outcomes according to different oocyte degeneration rates.*:*P<*0.05 compared with the other two groups, #*P<*0.05 compared with the other three groups. *P*<0.05 for the Bonferroni-adjusted following pairwise comparisons.

**Figure 3:**
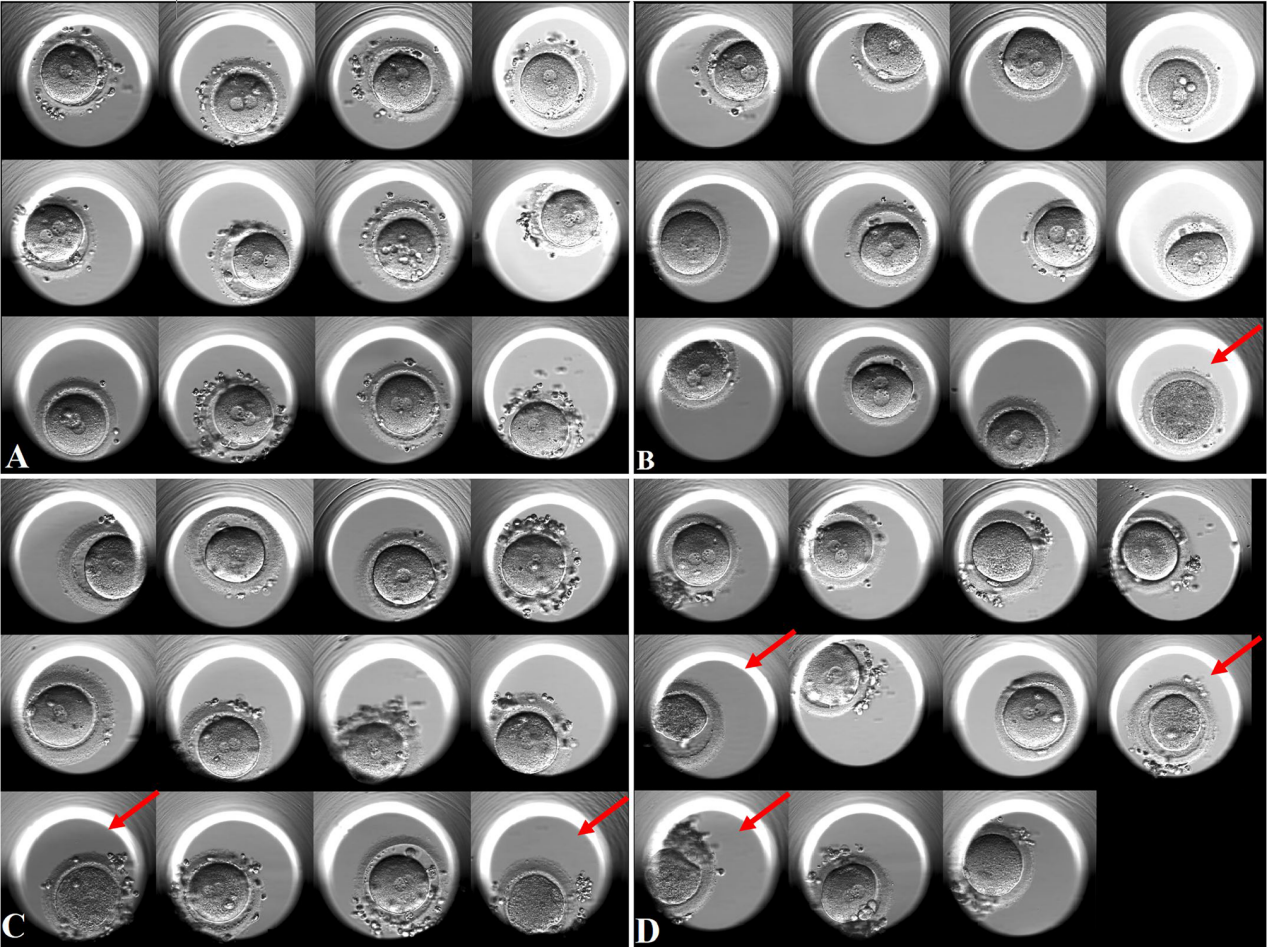
Oocyte images on Day 1 with different oocyte degeneration rates (ODR: 0, <10%, 10-20%, and >20%). Red arrow presents degenerated oocytes. **(A)** non-OD group(ODR 0). **(B)** ODR (<10%) subgroup. **(C)** ODR (10-20%) subgroup. **(D)** ODR (>20%) subgroup.

**Figure 4:**
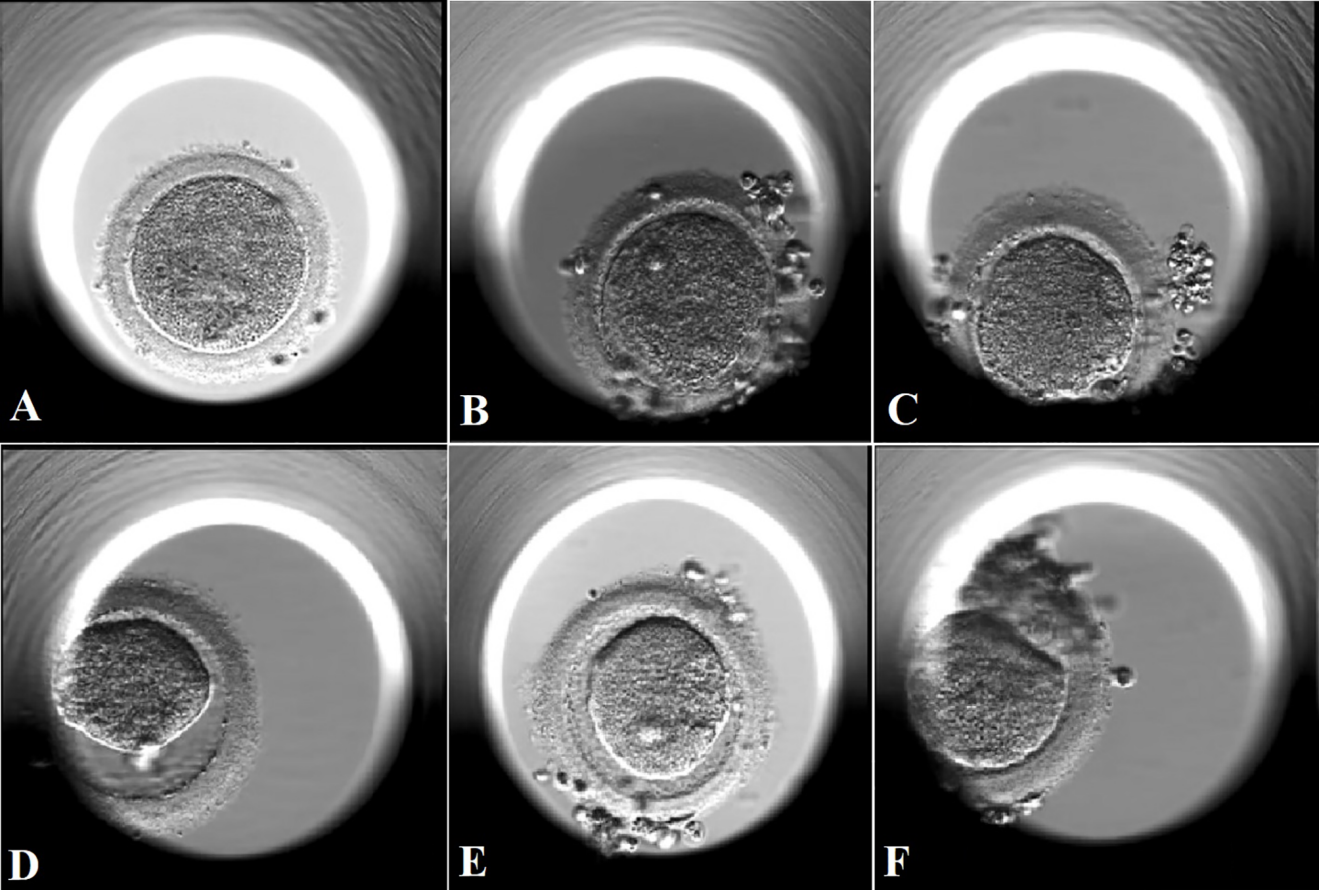
Amplified images of degenerated oocytes on Day 1 with different oocyte degeneration rates (ODR: 0, <10%, 10-20%, and >20%). **(A)**: amplified image of a degenerated oocyte in subgroup with ODR<10% in **(B)**. The oocyte was degenerated immediately after withdrawal of the injection needle, coexisting with sudden breakage upon needle entry and leakage of cytoplasm which led to the absence of PVS. **(B, C)**: amplified images of degenerated oocytes in subgroup with ODR 10-20% in **(C)**. **(D, F)**: amplified images of degenerated oocytes in in subgroup with ODR>20% in **(D)**. These oocytes were﻿ degenerated within a few hours after ICSI manipulation with shrank and darkened ooplasm.

Subgroup analysis according to different oocyte degeneration rate (non-OD group, ODR:<10%, 10-20%, and >20%) was shown in **Supplemental Table 3** and **Figure 2B**. The normal cleavage rate, blastocyst formation rate and live birth rate per OPU cycle were comparable among the four subgroups. Oocyte images and amplified oocyte images on Day 1 with different oocyte degeneration rate (ODR: 0, <10%, 10-20%, and >20%) were shown in **Figures 3**, **4**.

Nine technicians with different years of experience in ICSI micromanipulation performed the ICSI procedure in this study. The oocyte degeneration rate varied from 2.3% to 7.3%. Significant differences in oocyte degeneration rates were found among these technicians, as shown in **Figure 2A** and **Supplemental Table 2** (*P*=0.000). The 9^th^ technician possessed the lowest oocyte degeneration rate, with significant differences in comparison to the 1^st^, the 3^rd^, the 5^th^, and the 7^th^ technician. The normal fertilization rate and normal cleavage rate were also statistically different among these technicians (*P*=0.000, *P*=0.001 respectively) as shown in **Supplemental Table 2**. However, the blastocyte formation rate was statistically comparable among these technicians (*P*=0.677). As shown in **Supplemental Table 3**, proportions of the nine technicians were statistically comparable among different subgroups, except the 5^th^ technician.

Multivariable logistic regression analysis was performed to address the impact of any confounding variables that may account for oocyte degenerated (OD) after ICSI. As seen in **Table 2**, initial gonadotropin dosage (adjusted OR 1.006, 95% CI 1.001-1.010, *P*=0.013), E_2_ level on hCG day (adjusted OR 1.000, 95% CI 1.000-1.000, *P*=0.031), and the number of matured oocytes (adjusted OR 1.156, 95% CI 1.038-1.287, *P*=0.008) appeared to be independent risk factors for OD, after adjustment for female age, female BMI, duration of gonadotropin administration, FORT, number of retrieved oocytes and technicians.

**Table 2:** Multiple logistic regression analysis of oocyte degeneration to account for confounding variables.

Oocyte degeneration	Adjusted OR (95% CI)	Adjusted *P*
Female age (y)	0.995 (0.936-1.057)	0.862
Female BMI (kg/m2)	0.993 (0.925-1.066)	0.850
Initial gonadotropin dosage	1.006 (1.001-1.010)	0.013
Duration of gonadotropin administration	1.040 (0.929-1.164)	0.498
E2 level on hCG day, pg/ml	1.000 (1.000-1.000)	0.031
FORT	1.086 (0.842-1.402)	0.524
No. of retrieved oocytes	0.921 (0.827-1.026)	0.135
No. of matured oocytes	1.156 (1.038-1.287)	0.008
Technician	1.055 (0.976-1.140)	0.178

### Pregnancy Outcomes in Fresh Embryo Transfer Cycles

As evident in **Table 3**, there was no concurrent reduction in the incidence of live birth with the increasing of oocyte degeneration rate (ODR) (0, <10%, 10-20%, and >20%). Of note, the adjusted odds ratio of live birth rate per OPU cycle with oocyte degenerated (ODR subgroup <10%, 10-20%, and >20%) were comparable to the referent non-OD subgroup (ODR =0%) when adjusted for female age, female BMI, total gonadotropin dosage, E_2_ level and endometrium thickness on hCG day, number of oocytes retrieved, number of matured oocytes, number of normal fertilized and cleavage embryos, different technicians, suggesting an ODR-independent effect (adjusted OR 0.494, 0.661, 0.485, adjusted *P*=0.105, 0.392, 0.131).

**Table 3:** Odds of live birth rate per OPU cycle with increasing oocyte degeneration rate.

Comparison	OR (95% CI)	*P*	Adjusted	Adjusted *P*
			OR (95% CI)	
Referent group ODR=0 (N = 324)				
ODR <10% (N = 70) *vs* referent	0.710 (0.423-1.191)	0.193	0.494 (0.210-1.160)	0.105
ODR 10-20% (N = 68) *vs* referent	1.141 (0.669-1.945)	0.628	0.661 (0.256-1.705)	0.392
ODR >20% (N =26) *vs* referent	0.644 (0.289-1.436)	0.279	0.485 (0.190-1.240)	0.131

ODR, oocyte degeneration rate.

### Pregnancy Outcomes in FET Cycles

Pregnancy outcomes of FET cycles with or without oocyte degeneration in all transfer attempts are presented in **Supplemental Table 4**. In the OD group, 77 cycles achieved live births in fresh ET cycles, and 18 cycles had no more embryos after failed fresh ET cycles. Therefore, 80 FET cycles with vitrified embryos from 69 oocyte retrieval cycles were analyzed. In the non-OD group, 139 cycles achieved live births in fresh ET cycles, and 32 cycles had no more embryos after failed fresh ET cycles. Lastly, a total of 180 FET cycles with vitrified embryos from 153 oocyte retrieval cycles were analyzed (**Supplemental Table 4**). The proportion of cycles which did not return for FET was 9.2% in non-OD group, compared with 11.6% in OD group (*P*=0.573).

As presented in **Supplemental Table 4**, the ongoing pregnancy/live birth rate per transfer in FET cycles was not significantly different between OD and non-OD groups (38.8% *vs.* 43.9%; *P*=0.439). The overall miscarriage rate in FET cycles was slightly higher in the OD group (22.5% *vs.* 9.1%; *P*=0.038).

### Cumulative Pregnancy Outcomes

The cumulative live birth rate per OPU cycle was comparable between the OD group and non-OD group (63.4% *vs* 64.8%, *P*=0.760). There was no significant difference after adjusting for the following factors, female age, female BMI, total gonadotropin dosage, E_2_ level and endometrium thickness on hCG day, number of oocytes retrieved, number of matured oocytes, number of normal fertilized and cleavage embryos, and different technicians (**Table 4**).

**Table 4:** Cumulative live birth rate with or without oocyte degeneration in all transfer attempts.

Variable	None-OD group	OD group	OR	*P*	Adjusted OR	Adjusted *P*
	N = 324	N = 164	(95% CI)		(95% CI)	
Cumulative LBR per OPU cycle	210/324 (64.8)	104/164 (63.4)	1.063 (0.719-1.571)	0.760	0.776 (0.348-1.730)	0.536
Cumulative OPR/LBR per OPU cycle	218/324 (67.3)	108/164 (65.9)	1.066 (0.717-1.587)	0.751	0.928 (0.411-2.093)	0.856

OP/LBR, ongoing pregnancy/live birth rate.

## Discussion

Our study provides cycle-based evidence that the presence of oocyte degeneration after ICSI is not an indicator for predicting the cumulative live birth rate per OPU cycle in young women. Initial gonadotropin dosage, E_2_ level on hCG day and the number of matured oocytes appeared to be independent risk factors for oocyte degeneration.

Previous studies suggested that oocyte degeneration be associated with ovarian stimulation. Vigorous ovarian stimulation, presented with more gonadotropin dosage and higher E_2_ level on hCG day, would have a greater detrimental effect on fertilization and the number of good quality embryos. Theoretically, E_2_ level on hCG day is a marker of granulosa cell function as well as ovarian response, directly affecting oocyte maturation during controlled ovarian stimulation. In the study of Xia et al., degeneration rates were higher in cases where the total number of oocytes retrieved was greater than 20 and the E_2_ was >3,000 pg/mL on hCG day (18). However, Rosen et al. (12) reported that the E2 on hCG day (patient response) was negatively associated with degeneration rate in women who received down-regulated protocols. Palermo et al. (7 *)* also reported that oocyte degeneration and fragile membranes were increased in patients who had more gonadotropins administered and lower E2 levels on hCG day. These contradictory findings may due to different stimulation protocols (down-regulated or antagonist protocols), incomparable baseline E_2_ levels of enrolled patients, and heterogeneity in the cohort of retrieved oocytes (as demonstrated by higher oocyte maturation rate) in those studies. In our clinics, controlled stimulation protocols for young patients with good prognosis are mainly mid-luteal phase long protocol and antagonist protocol, which approximately account for 60% and 40% respectively. As shown in **Table 1**, different proportions of ovarian stimulation protocols, including mid-luteal phase long protocol and antagonist protocol, were statistically comparable between OD and non-OD groups (*P*=0.259). The results of our study were in accordance with the previous studies showing oocyte degeneration appears to be a function of patients’ response (7, 12). Both E_2_ level on hCG day and the number of matured oocytes were independent risk factors for oocyte degeneration in our study. It is worthy to emphasize that our study only included young patients with normal response.

Previous studies provided evidence that oocyte degeneration rate is independent of the ICSI technician in experienced hands (12, 13). Although ICSI is a delicate procedure requiring considerable skills and experience by laboratory technicians, there are few publications sustaining the idea that oocyte degeneration rate may be a function of technician skills (19, 20). A total of 9 technicians with different years of experience performed ICSI in our center. Significant differences in oocyte degeneration rate, normal fertilization rate and cleavage rate were found among these technicians, which maybe explain by learning curve of technicians. However, blastocyst formation rate was statistically comparable, providing evidence of competence of embryo development even with different oocyte degeneration rates. Besides, confounding variables including technician have been adjusted.

The removal of the cumulus cells from the oocyte prior to ICSI allows better observation of oocyte morphology. The impact of all these oocyte features, including the granularity of the cytoplasm, the morphology of the first polar body, and thickness of zona pellucida, on the oocyte degeneration rate is still controversial (21–23). If the diameter of the cumulus-removal pipette is too small, then oocytes can be strongly compressed when aspirated into the pipette, possibly resulted in fragmented polar bodies and “crater” like cytoplasm, favoring the leaking away of the cytoplasm after injection, thus resulting in degeneration. In addition, the first polar body morphology is a ‘fragile’ parameter because it is susceptible to female ageing. Therefore, it was suggested that using the first polar body morphology as primary prognostic factor of oocyte quality and reproductive outcome was hazardous (24). The morphology of the zona pellucida observed under light microscopy seems not to be correlated with normal fertilization and embryo development (25, 26). ﻿During the denudation procedure, it occasionally happened that zona pellucida was damaged, resulting in the partial or total extrusion of the ooplasm out of the zona pellucida. However, commercialized denuding pipette was used in our lab, which significantly lowered the chance of mechanical damage during denudation. Nevertheless, we admit that we may need more careful observation in the future.

Oocyte degeneration may reduce oocyte utilization rate. In our study, normal fertilization rate and number of normal cleavage embryos were significantly lower in OD group. Besides, the normal fertilization rate presented a decline tendency with significant differences in different ODR subgroups (0, <10%, 10-20%, >20%). There were fewer number of good quality embryos and available embryos in the OD group compared to the non-OD group (3.44±2.23 *vs* 4.05±2.51, *P*=0.009; 4.41±2.13 *vs* 4.87±2.31, *P*=0.032). The compromised fertilization rate, lower number of available and good quality embryos, and lower number of vitrified blastocysts in OD group may result from oocyte degeneration, resulting in the reduction of the numbers of survived oocytes and embryos. However, for a cohort of oocytes retrieved from the same ICSI treatment cycle, whether the presence of oocyte degeneration can reflect the sibling oocyte quality and predict the sibling embryo development potential and clinical pregnancy outcome remains controversial. Theoretically, there is heterogeneity in the growing follicle cohort with some follicles better than others, therefore, producing oocytes that have different developmental competence. Heterogeneity of follicles may explain non-uniform behavior of the oolemma during the injection (11), which suggests that not all oocytes are “equal” and their fate may be established by preexisting conditions before retrieval to some extent. Whether the phenomenon of oocyte degeneration reflects lower competence of sibling oocytes remains a question. Previous studies (11, 12, 18) showed that fertilization ability after ICSI of fragile oocytes was lower than that of normal oocytes, but the resultant embryos had the same developmental ability as those of normal oocyte-derived embryos. In our study, even with lower number of cleavage stage embryos for blastocyst culture, blastocyst formation rate was comparable between OD group and non-OD group in our study, suggesting the uncompromised development capacity of sibling embryos, which was similar to the results of previous studies. Moreover, it is worthy to note that the live birth rate in fresh cycles did not change with the increasing oocyte degeneration rate after adjusting confounding variables. In addition, clinical pregnancy rate, ongoing pregnancy/live birth rate per transfer in FET cycles were not significantly different between the OD and non-OD groups. The results demonstrated that the presence of oocyte degeneration is not an indicator to predict the likelihood of live birth rate in fresh ICSI cycles and subsequent FET cycles.

For clinicians, cumulative live birth rate per OPU cycle provides a more meaningful and comprehensive estimate of treatment success in ART in its totality as cryopreservation has become an integral part of ART. Currently, there is no consensus on the preferred method or criteria for presenting the cumulative live birth rate following ART treatment (27, 28). One critical aspect of calculating the cumulative LBR is how to deal with couples who do not return for ART treatment in the analysis. Our study showed a similar proportion of OPU cycles which did not return for FET between OD and non-OD group. Thus, women who failed to return for FET (either because they moved to another IVF center or stopped treatment for any other reason) were also included in the cumulative LBR as the denominator. We considered that women should be censored after their first live birth, hence only including the first live birth in the numerator. To make the results relevant to all women referred for their ICSI treatment, we used all OPU cycles as the denominator. The period of follow-up also plays an important role on computing cumulative live birth rates. Maheshwari et al. proposed a three-step approach to report short, medium and long-term cumulative livebirth rates (27). We enrolled patients from January 2018 and they were followed from their first fresh ICSI cycles either to the first live birth or until November 2020, regardless of whether all frozen embryos had been transferred. Thus, our study had a follow-up time of 2 years. In an intent-to-treat analysis, no significant difference was found in the cumulative live birth rate per fresh OPU cycle between the oocyte degeneration group and non-oocyte degeneration group. After adjusting the confounding factors, the cumulative LBR per fresh OPU cycle remains statistically comparable between the two groups (95% CI 0.348-1.730; *P*=0.536). It is worth noting that although there were fewer numbers of good quality embryos and available embryos in the OD group compared to the non-OD group (3.44±2.23 *vs* 4.05±2.51,*P*=0.009; 4.41±2.13 *vs* 4.87±2.31,*P*=0.032), this did not influence the cumulative LBR per OPU cycle, even after adjusting confounding factors. Our findings indicate that the presence of oocyte degeneration in ICSI cycles is not an indicator for prediction of cumulative LBR per OPU cycle.

This is to our knowledge the first study comparing cumulative LBR in oocyte degeneration group and non-oocyte degeneration group. The main strength of the current study is its demonstration that the presence of oocyte degeneration in ICSI cycles cannot be used to predict cumulative LBR per OPU cycle. The formulation of this conclusion was based on the pregnancy outcomes including fresh cycles and subsequent FET cycles, and was reinforced by multivariable logistic regression analysis to account for potential confounding variables. Despite these strengths, we also acknowledge some limitations. First, the time interval for the follow-up was not long enough for all the frozen embryos to be transferred. Moreover, the retrospective nature of the study introduces the potential to include confounding variables that may bias our results, although we performed multiple logistic regression analysis to minimize these effects.

In conclusion, our results provide cycle-based evidence that the presence of oocyte degeneration after ICSI is not an indicator for predicting the cumulative live birth rate per OPU cycle in young women.

## Data Availability Statement

The raw data supporting the conclusions of this article will be made available by the authors, without undue reservation.

## Ethics Statement

The studies involving human participants were reviewed and approved by Reproductive Medicine Center, The First Affiliated Hospital of Sun Yat-sen University. The patients/participants provided their written informed consent to participate in this study.

## Author Contributions

YX supervised the entire study, including the procedures, conception, design and completion. YW and PL were responsible for the collection of data. XH contributed to the data analysis and drafted the article. CZ participated in the interpretation of the study data and in revisions to the article. YL and XZ and CD were responsible for ICSI micromanipulation. All authors contributed to the article and approved the submitted version.

## Conflict of Interest

The authors declare that the research was conducted in the absence of any commercial or financial relationships that could be construed as a potential conflict of interest.

## Publisher’s Note

All claims expressed in this article are solely those of the authors and do not necessarily represent those of their affiliated organizations, or those of the publisher, the editors and the reviewers. Any product that may be evaluated in this article, or claim that may be made by its manufacturer, is not guaranteed or endorsed by the publisher.

## Funding

National Key Research and Development Program (2018YFC1003102), Natural Science Foundation of Guangdong Province (2018A030313789) and Guangdong Province Key Laboratory of Reproductive Medicine (2012A061400003).
