# Supplementary material for: Oocyte Degeneration After ICSI Is Not an Indicator of Live Birth in Young Women
Source: Front Endocrinol (Lausanne). 2021 Aug 16;12:705733. doi: 10.3389/fendo.2021.705733 (PMC8415476; doi:10.3389/fendo.2021.705733)
Supplement: Supplementary file 1 [file Table_1.docx]

**Supplemental Table1** Baseline characteristics and reproductive outcomes of all fresh cycles

| Characteristic | Fresh cleavage embryo transfer N=488 |
| --- | --- |
| Female age (y) | 30.62$\pm$3.72 |
| Male age (y) | 33.42$\pm$5.97 |
| History of infertility | 3.70$\pm2.45$ |
| Basal FSH, IU/L | 5.68$\pm1.37$ |
| Basal LH, IU/L | 3.35$\pm1.88$ |
| Basal E2, pg/ml | 31.14$\pm19.50$ |
| Female BMI (kg/m^2^) | 21.30$\pm2.84$ |
| Total gonadotropin dosage, IU | 2286.55$\pm838.86$ |
| LH level on hCG day, IU/L | 1.19$\pm1.11$ |
| E2 level on hCG day, pg/ml | 2533.17$\pm949.79$ |
| P level on hCG day, | 0.72$\pm0.26$ |
| Endometrial thickness on hCG day (mm) | 11.36$\pm2.72$ |
| Stimulation protocol |  |
| mid-lutealphase long protocol | 341 |
| antagonist protocol | 147 |
| FORT | 53.70(3112/5795) |
| Oocyte maturation rate | 81.65(5285/6473) |
| Oocyte degeneration rate | 4.64(245/5285) |
| Normal fertilization rate | 76.76(4057/5285) |
| Normal cleavage rate | 97.02(3936/4057) |
| Blastocyte formation rate | 59.72(1533/2567) |
| No. of available embryos | 4.72$\pm$2.26 |
| No. of good quality embryos | 3.84$\pm2.43$ |
| No. of transferred D3 embryos | 1.92$\pm0.28$ |
| Implantation rate | 36.22(339/936) |
| Biochemical rate | 4.30(21/488) |
| Miscarriage rate | 11.86(30/253) |
| Ectopic pregnancy rate | 1.98(5/253) |
| Clinical pregnancy rate | 51.84(253/488) |
| Live birth rate/per OPU cycle | 44.26(216/488) |
| Multiple pregnancy rate | 25.30(64/253) |
| Mode of delivery |  |
| Cesarean | 58.33 (126/216) |
| Vaginal | 41.67(90/216) |
| Sex of newborn |  |
| Boy | 50.36 (141/280) |
| Girl | 49.64 (139/280) |
| Gestational age (wk) |  |
| $\geq$37 | 76.85 (166/216) |
| $<$34 | 5.09 (11/216) |
| $\geq34$,$<37$ | 18.06 (39/216) |

Note: Values are mean$\pm$SD or percentage (number); BMI: body mass index; E2: estradiol; FSH: follicle stimulating Hormone; LH: luteinizing hormone; FORT: follicle output rate.
