# Supplementary material for: Oocyte Degeneration After ICSI Is Not an Indicator of Live Birth in Young Women
Source: Front Endocrinol (Lausanne). 2021 Aug 16;12:705733. doi: 10.3389/fendo.2021.705733 (PMC8415476; doi:10.3389/fendo.2021.705733)
Supplement: Supplementary file 2 [file Table_2.docx]

**Supplemental Table 2** Oocyte degeneration rates and embryo development among ICSI technicians

| Technician | 1 | 2 | 3 | 4 | 5 | 6 | 7 | 8 | 9 | *P* value |
| --- | --- | --- | --- | --- | --- | --- | --- | --- | --- | --- |
| No. of years of ICSI | 2 | 3 | 3 | 4 | 5 | 5 | 6 | 11 | 11 |  |
| Degeneration rate (%) | 5.6*  (37/664) | 3.4  (24/711) | 7.3*  (40/547) | 3.8  (19/505) | 5.9*  (50/850) | 4.0  (20/506) | 5.9*  (29/492) | 3.6  (7/194) | 2.3  (19/816) | 0.000 |
| Normal fertilization rate(%) | 70.3  (467/664) | 82.1#  (584/711) | 74.0  (405/547) | 70.9  (358/505) | 78.4#  (666/850) | 75.3  (381/506) | 79.3#  (390/492) | 78.9  (153/194) | 80.0#  (653/816) | 0.000 |
| Normal cleavage rate(%) | 94.6  (442/467) | 97.6#  (570/584) | 99.0#  (401/405) | 98.9  (354/358) | 96.2  (641/666) | 95.3  (363/381) | 97.7  (381/390) | 97.4  (149/153) | 97.2  (635/653) | 0.001 |
| Blastocyte formation rate(%) | 58.4  (174/298) | 62.6  (251/401) | 58.3  (126/216) | 63.4  (151/238) | 56.7  (234/413) | 58.8  (130/221) | 61.6  (154/250) | 56.2  (59/105) | 59.8  (254/425) | 0.677 |

Note:*:*P*$<$0.05 compared with the 9^th^ technician. #: *P*$<$0.05 compared with the 1^st^ technician. The 2^nd^ technician possessed the highest normal fertilization rate, with significant differences in comparison to the 1^st^, the 3^rd^, and the 4^th^ technician.
