# Supplementary material for: Oocyte Degeneration After ICSI Is Not an Indicator of Live Birth in Young Women
Source: Front Endocrinol (Lausanne). 2021 Aug 16;12:705733. doi: 10.3389/fendo.2021.705733 (PMC8415476; doi:10.3389/fendo.2021.705733)
Supplement: Supplementary file 3 [file Table_3.pdf]

Supplemental Table 3 Effect of oocyte degeneration rate on laboratory and ICSI cycle outcomes in fresh embryo transfer cycles

	Oocyte degeneration rate (%)				*P*
	0(N=324)	<10(N=70)	10-20(N=68)	>20(N=26)	
Female age (y)	30.5±3.7	30.9±3.6	30.6±4.0	31.5±4.0	0.551
Technician					
1	9.3(30/324)	20.0(14/70)	14.7(10/68)	11.5(3/26)	0.068
2	13.6(44/324)	14.3(10/70)	11.8(8/68)	0(0/26)	0.239
3	10.8(35/324)	5.7(4/70)	14.7(10/68)	19.2(5/26)	0.192
4	9.9(32/324)	8.6(6/70)	5.9(4/68)	11.5(3/26)	0.735
5	16.7(54/324)	10.0(7/70)	14.7(10/68)	38.5(10/26)	0.010
6	10.8(35/324)	12.9(9/70)	7.4(5/68)	3.8(1/26)	0.493
7	7.7(25/324)	12.9(9/70)	13.2(9/68)	15.4(4/26)	0.240
8	3.7(12/324)	2.9(2/70)	5.9(4/68)	0(0/26)	0.561
9	17.6(57/324)	12.9(9/70)	11.8(8/68)	0(0/26)	0.071
No. of available embryos	4.9±2.3	5.4±2.4	3.8±1.7@	3.4±1.2@	0.000
No. of good quality embryos	4.0±2.5	4.4±2.3	2.9±2.0@	2.3±1.3@	0.000
Follicle output rate	51.2%(2009/3925)	61.1(502/821)*	57.1(445/780)	58.0(156/269)	0.000
No. of retrieved oocytes	13.1±3.3	14.7±2.9#	13.0±3.7	11.9±2.6	0.000
No. of matured oocytes	10.5±3.3	13.0±2.6#	10.4±3.4	9.7±2.3	0.000
Oocyte maturation rate	80.3(3412/4248)	88.5(911/1029)#	80.0(709/886)	81.6(253/310)	0.000
Normal fertilization rate	80.8(2758/3412)#	71.7(653/911)	70.1(497/709)	58.9(149/253)&	0.000
Normal cleavage rate	97.1(2679/2758)	97.5(637/653)	95.6(475/497)	97.3(145/149)	0.221
Blastocyst formation rate	59.9(1073/1791)	63.0(279/443)	54.7(152/278)	52.7(29/55)	0.110
LBR	42.9(139/324)	51.4(36/70)	39.7(27/68)	53.8(14/26)	0.356

Note: Values are mean±SD or percentage (number), $P<0.05$ for the Bonferroni-adjusted following pairwise comparisons, LBR: live birth rate per OPU cycle; @: $P<0.05$ compared with the other two groups, *:$P<0.05$ compared with the non-OD group (oocyte degeneration rate=0);#:$P<0.05$ compared with the other three groups;&:$P<0.05$ compared with the other three groups.
