# Supplementary material for: Oocyte Degeneration After ICSI Is Not an Indicator of Live Birth in Young Women
Source: Front Endocrinol (Lausanne). 2021 Aug 16;12:705733. doi: 10.3389/fendo.2021.705733 (PMC8415476; doi:10.3389/fendo.2021.705733)
Supplement: Supplementary file 4 [file Table_4.docx]

**Supplemental Table 4** Pregnancy outcomes of FET cycles with or without oocyte degeneration in all transfer attempts

| Variable | None-OD group  N=153 | OD group  N=69 | *P* |
| --- | --- | --- | --- |
| No. of OPU cycles not return for FET^*^ | 14 | 8 | 0.573 |
| Total No. of FET cycles | 180 | 80 | - |
| No. of FET attempts |  | | |
| 1 | 104 | 45 | - |
| 2 | 30 | 26 | - |
| 3 | 4 | 9 | - |
| 4 | 1 | 0 | - |
| Implantation rate | 43.7(80/183) | 44.4(44/99) | 0.906 |
| Miscarriage rate | 9.1(8/88) | 22.5(9/40) | 0.038 |
| Clinical pregnancy rate per transfer | 48.9(88/180) | 50.0(40/80) | 0.869 |
| Live birth rate per transfer | 39.4(71/180) | 33.8(27/80) | 0.382 |
| OPR/LBR per transfer | 43.9(79/180) | 38.8(31/80) | 0.439 |

Note: Values are percentage (number); OPR/LBR: on going pregnancy rate/live birth rate; *: the number of cycles eligible to return for FET cycle with frozen embryos who failed to get live births in fresh ET cycles.
